# Genomic Diversity and Evolution of the Lyssaviruses

**DOI:** 10.1371/journal.pone.0002057

**Published:** 2008-04-30

**Authors:** Olivier Delmas, Edward C. Holmes, Chiraz Talbi, Florence Larrous, Laurent Dacheux, Christiane Bouchier, Hervé Bourhy

**Affiliations:** 1 Institut Pasteur, UPRE Lyssavirus Dynamics and Host Adaptation, World Health Organization Collaborating Centre for Reference and Research on Rabies, Paris, France; 2 Mueller Laboratory, Center for Infectious Disease Dynamics, Department of Biology, The Pennsylvania State University, University Park, Pennsylvania, United States of America; 3 Institut Pasteur, Plate-forme Génomique - Pasteur Genopole® Ile de France, Paris, France; 4 Fogarty International Center, National Institutes of Health, Bethesda, Maryland, United States of America; University of Oxford, United Kingdom

## Abstract

Lyssaviruses are RNA viruses with single-strand, negative-sense genomes responsible for rabies-like diseases in mammals. To date, genomic and evolutionary studies have most often utilized partial genome sequences, particularly of the nucleoprotein and glycoprotein genes, with little consideration of genome-scale evolution. Herein, we report the first genomic and evolutionary analysis using complete genome sequences of all recognised lyssavirus genotypes, including 14 new complete genomes of field isolates from 6 genotypes and one genotype that is completely sequenced for the first time. In doing so we significantly increase the extent of genome sequence data available for these important viruses. Our analysis of these genome sequence data reveals that all lyssaviruses have the same genomic organization. A phylogenetic analysis reveals strong geographical structuring, with the greatest genetic diversity in Africa, and an independent origin for the two known genotypes that infect European bats. We also suggest that multiple genotypes may exist within the diversity of viruses currently classified as ‘Lagos Bat’. In sum, we show that rigorous phylogenetic techniques based on full length genome sequence provide the best discriminatory power for genotype classification within the lyssaviruses.

## Introduction

Lyssaviruses (LYSSAV) are RNA viruses with single-stranded, negative-sense genomes of the family *Rhabdoviridae* [1] that infect a variety of mammals causing rabies-like diseases. Rabies is an ancient disease that may have been reported in the Old World before 2300 B.C. [2]. However, the absence of effective control measures in animal reservoir populations combined with a widespread lack of human access to vaccination means that more than 50,000 people annually die of rabies, particularly in Asia and Africa [3], [4]. Currently, there are seven recognised genotypes (GT) of LYSSAV defined on the basis of their genetic similarity [5], [6]: rabies virus (RABV, GT1) responsible for classical rabies in terrestrial mammals globally and in bats on the American continent, as well as the cause of most rabies-related human deaths worldwide [3]; Lagos bat virus (LBV, GT2); Mokola virus (MOKV, GT3); Duvenhage virus (DUVV, GT4); European bat lyssavirus type 1 (EBLV-1, GT5); European bat lyssavirus type 2 (EBLV-2, GT 6); and Australian bat lyssavirus (ABLV, GT7). All genotypes except MOKV (where the host species is unknown) have bat reservoirs, hinting that lyssaviruses originated in these mammals [7]. Additionally, four new lyssavirus genotypes that infect bats in central and southeast Asia have been proposed: Aravan virus, Khujand virus, Irkut virus and West Caucasian Bat virus [8], [9]. The negative-sense LYSSAV genome encodes five proteins: the nucleoprotein (N), phosphoprotein (P), matrix protein (M), glycoprotein (G) and RNA polymerase (L) in the order 3′-N-P-M-G-L-5′ [10].

Despite the importance of LYSSAV for human and wildlife populations, the number of complete genome sequences of field isolates of LYSSAV is sparse, with only eight currently available for limited type species [11]–[15]. Herein, we present the first genomic and evolutionary analysis of the seven known genotypes of LYSSAV, therein significantly increasing the extent of available genome sequence data available for these important mammalian pathogens.

## Materials and Methods

### Viruses and RNA isolation

Total RNA (Table 1) was isolated from original specimens or from suckling mice brain after early passage using Tri-Reagent (Euromedex). The only exception was the 8743THA isolate that was adapted on BSR cells (passage 22). For this isolate, total RNA was isolated from infected BSR cells infected at a low multiplicity of infection (0,1). Reverse transcription was performed with random hexamer primer (Roche Boehringer) using Superscript II (Invitrogen) following the manufacturer instructions.

**Table 1:** Isolates of lyssavirus analysed in this study.

Genus and name	Reference no.	Host species/vector	Origin	Year of first isolation	GenBank accession no.
**Lyssavirus Genotype 1**					
Rabies virus	8743THA	Human	Thailand	1983	EU293121
Rabies virus	8764THA	Human	Thailand	1983	EU293111
Rabies virus	9147FRA	Fox	France	1991	EU293115
Rabies virus	9001FRA	Dog bitten by a bat	French Guyana	1990	EU293113
Rabies virus	9704ARG	Bat (*Tadarida brasiliensis*)	Argentina	1997	EU293116
Rabies virus	SHBRV-18	Bat (*L. noctivagans*)	USA	1983	AY705373
Rabies virus	NNV-RAB-H	Human	India	2006	EF437215
Rabies virus	RABV	Human	India	2004	AY956319
Rabies virus	SADB19	Vaccine			M31046
Rabies virus	PV	Vaccine			NC_001542
**Genotype 2**					
Lagos bat virus	8619NGA	Bat (*Eidolon helvum*)	Nigeria	1956	EU293110
Lagos bat virus	0406SEN	Bat (*Eidolon helvum*)	Senegal	1985	EU293108
**Genotype 3**					
Mokola virus	MOKV	Cat	Zimbabwe	1981	NC_006429
Mokola virus	86100CAM	Shrew	Cameroun	1974	EU293117
Mokola virus	86101RCA	Rodent	RCA	1981	EU293118
**Genotype 4**					
Duvenhage virus	94286SA	Bat (*Minopterus sp.*)	South Africa	1981	EU293120
Duvenhage virus	86132SA	Human	South Africa	1971	EU293119
**Genotype 5**					
European bat lyssavirus 1	8918FRA	Bat (*Eptesicus serotinus*)	France	1989	EU293112
European bat lyssavirus 1	03002FRA	Bat (*Eptesicus serotinus*)	France	2003	EU293109
European bat lyssavirus 1	RV9	Bat (*Eptesicus serotinus*)	Germany	1968	EF157976
**Genotype 6**					
European lyssavirus 2	9018HOL	Bat (*Myotis dasycneme*)	Holland	1986	EU293114
European lyssavirus 2	RV1333	Human	Scotland	2002	EF157977
**Genotype 7**					
Australian bat lyssavirus	ABLh	Human	Australia	1986	AF418014
Australian bat lyssavirus	ABLb	Bat (*Pteropus species*)	Australia	1996	NC_003243

Genbank accession numbers for the newly acquired sequences are designated EU293108-EU293121.

### PCR and sequence determination

Long-range PCR products were obtained using ExTaq (Takara) and specific primers (Table S1) using manufacturer recommendations. For sequence determination we used a shotgun base approach called LoPPS (Long PCR Product Sequencing) [16], [17]. 3′ genomic ends were generated by RACE protocol [14] using a 5′ phosphorylated reverse complementary T7 primer. T7 cDNAs were further used for heminested-PCR with ExTaq using T7 and two strain specific primers designed in the N coding region (supplementary Table 1). To determine the 5′ sequence of the genomic RNA we used a 5′RACE version 2.0 kit from Invitrogen following manufacturer instructions. The PCR products (5′ or 3′ RACE) were then purified on gel using Qiaquick gel extraction kit (Qiagen) and cloned in PCR 2.1 TOPO T/A (Invitrogen) for sequencing. Each position of the consensus nucleotide sequence was determined from at least three independent sequences. All consensus sequences obtained using Sequencher 4.7 (Gene Codes) software were aligned using ClustalX 1.83.1 [18]. The untranslated regions were further aligned manually using the SE-AL program (http://tree.bio.ed.ac.uk/). GenBank accession numbers for the sequences newly acquired here are designated EU293108-EU293121.

### Phylogenetic analysis

Phylogenetic analysis of LYSSAV genomes was based on a multiple alignment of concatenated coding region sequences (12105 nt). A maximum likelihood (ML) phylogenetic analysis of these data was undertaken using PAUP* [19] employing the best-fit GTR+I+Γ_4_ model of nucleotide substitution inferred by ModelTest [20]. To determine the extent of support for different groupings on the tree a bootstrap resampling analysis was undertaken employing 1000 replicate neighbor-joining trees estimated under the ML substitution model.

## Results and Discussion

In total we determined 14 new complete genome sequences of field isolates representing six (GT1, GT2, GT3, GT4, GT5 and GT6) of the seven genotypes of LYSSAV, with complete genome from GT4 obtained for the first time. These genomes were combined with eight genomes described previously (with the exception of one Australian bat lyssavirus for which leader and trailer sequences are unavailable). Eight field isolates of viruses isolated from humans, canids and bats were chosen as representative of the diversity of GT1. Two vaccine strains (SAD-B19 and PV) were included in all sequence comparisons but not in the phylogenetic analysis. Our study also represent the first analysis of the intrinsic genetic diversity of GT2, GT3, GT4, GT5 and GT6 based on full length genomes.

**Figure 1:**
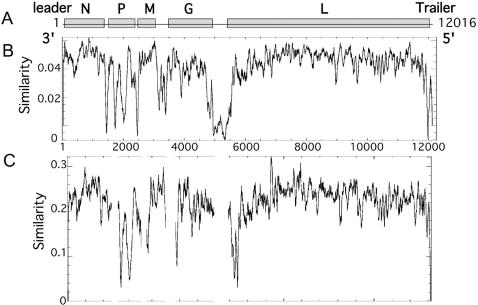
Schematic representation of lyssavirus genome organization and sequence similarity among 24 aligned genomes. A. The 3′ leader, N-, P-, M-, G- and L-coding regions and the 5′ trailer region are shown. B. Sequence similarity is calculated by moving a window of 60 nucleotides along the aligned sequences. C. Sequence similarity is calculated by moving a window of 20 amino acids along the aligned sequences. Within each window, the similarity of any one position is taken to be the average of all the possible pairwise scores at that position and is calculated using PLOTCON (available at http://bioweb.pasteur.fr/seqanal/interfaces/plotcon.html).

All genomes have the same structural organization although their lengths varied between 11918 nt. (GT7) and 12016 nt. (GT2) (Table 2). The predicted size of the coding regions is similar among genotypes, with the M protein identical in length across all genotypes and the P protein the most variable [14], [21], 22. As observed in other RNA viruses, all genotypes show a bias toward G+C richness [23], with the lowest G+C content observed in GT2 and the highest in GT1 (Table 2). All genomes have a polycistronic genome organization surrounded by untranslated regions (Table S2) similar to that already described [10], [14]. The extent of genetic diversity, reflected in percentage identity, varies within and among proteins (Figure 1), in the order N>L>M>G>P (95.2, 94.2, 92.3, 85.8, 81.5% amino acid identity, respectively). A similar pattern was previously observed using more limited data sets [14]. This same order was also observed in terms of overall selection pressure, measured as the mean ratio of nonsynonymous (d_N_) to synonymous substitutions (d_S_) per site (d_N_/d_S_), estimated using the maximum likelihood SLAC (Single Likelihood Ancestor Counting; http://www.datamonkey.org/) method [24]: N = 0.048; L = 0.055; M = 0.078; G = 0.119; P = 0.187. This approximately four-fold difference in mean d_N_/d_S_ reflects major differences in selective constraint among proteins. This trend was also reflected in previous analyses of full length genomes of vaccine strains [22] and through partial gene comparisons [25], [26].

**Table 2:** Coding potential, genome size (in nucleotides) and G+C content of 24 genomes representing the 7 genotypes of the lyssavirus genus.

Genotype	1	2	3	4	5	6	7	All genotypes
**3′UTR**	70	70	70	70	70	70	70	70
**N protein**	1353	1353	1353	1356	1356	1356	1353	1353–1356
**N-P**	90–94	101	100–102	90	90–96	101	93–94	90–102
**P protein**	894	918	912	897	894	894	894	894–918
**P-M**	87–90	75	80–83	83	83	88	87	75–90
**M protein**	609	609	609	609	609	609	609	609
**M-G**	211–215	204	203–204	191	211	205–210	207–209	191–215
**G protein**	1575	1569	1569	1602	1575	1575	1578–1581	1569–1602
**G-L**	515–525	578–588	546–562	562–563	560	511–512	508–509	508–588
**L protein**	6384–6429	6384	6381–6384	6384	6384	6384	6384–6387	6381–6429
**5′UTR**	86–131	145	112–114	131	130–131	131	131	86–145
**Genome**	11923–11928	12006–12016	11940–11957	11975–11976	11966–11971	11924–11930	11918	11918–12016
**G+C%**	44,9–45,4	40,9–43,5	44,1–44,9	44,1–44,2	44,6–45,0	44,8	43,4–44,2	40,9–45,4

Upper and lower size ranges are indicated.

Our study represents the largest analysis of the 3′ and 5′ UTR of the lyssavirus genomes undertaken to date. The 3′ UTR comprises 70 nt and includes the leader regions potentially transcribed into the leader RNA. The 5′UTR region comprises 86–145 nt and contains the trailer regions of size 68–69 nt. Both the 3′ and 5′ UTR have conserved signals that play a role to modulate replication and transcription (Figure S1) [27]. Our data also reveals a strict complementarity limited to the 9 terminal nucleotides as well as nucleotide positions 14 and 16 from both ends of the genome [14], [28], [29].

**Figure 2:**
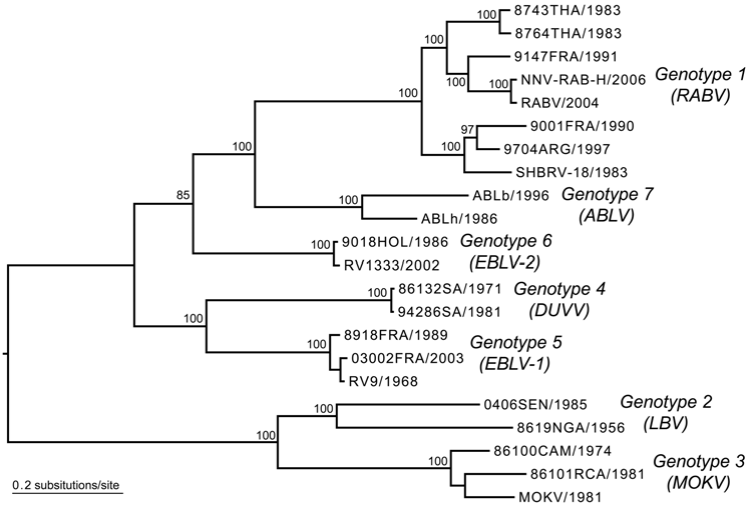
Phylogenetic relationships of 22 complete coding regions of LYSSAV genomes representatives of the 7 genotypes. The phylogeny was inferred using an ML procedure, and all horizontal branches are scaled according to the number of substitutions per site. Boot strap values (>95%) are shown for key nodes. The tree is mid-point rooted for purposes of clarity only.

There have been several attempts to estimate the evolutionary relationships among lyssaviruses, with most utilizing only one or two genes [1], [7], [21], [26], [30]–[34]. We therefore undertook a phylogenetic analysis of 22 genomes representative of the seven genotypes of LYSSAV based on a multiple alignment of concatenated coding sequences. Our phylogenetic analysis reveals the separation of LYSSAV into two major branches previously defined as different ‘phylogroups’ [7] and 7 component lineages defined as genotypes [5], [35]. Phylogroup 1 comprised GT1, 4, 5, 6 and 7, while phylogroup 2 contains only GT2 and GT3 (Figure 2). Notably, phylogroup 2 contains viruses of sampled exclusively from Africa – LBV and MOKV – while a third African genotype (DUVV) is found within phylogroup 1 [32], [36]. Also of note was the observation that although GT5 and GT6 both circulate in European insectivorous bats [32], the former is more closely related to the African GT4 viruses [32]. Hence, there has clearly been an independent origin of genotypes 5 and 6 in European bats, as previously documented in analyses of the N and G genes in isolation [32]. Finally, that bats appear as the principle host species across such a large phylogeographic range indicates that the association between lyssaviruses and bats is likely to be the ancestral condition (with a secondary loss of bat transmission in GT3), such that the movement of bats is likely to be responsible for the global dissemination of these viruses [7].

Notably, our study represents the first analysis of the genetic diversity of four complete genomes of GT2 and GT4, both of which are African in origin. While little variation is seen within GT4, the degree of divergence among the two GT2 isolates (Lagos bat virus – LBV) is striking (23.7% and 12.1% at the nucleotide and amino acid levels, respectively) and greater than that seen within any other genotype. Hence, although 0406SEN and 8619NGA are related according to the arbitrary classification system based on nucleotide identity between N coding regions (80.3% between 0406SEN and 8619NGA compared to a cut-off of 80%) [6], [21], this classification system will likely need to be revised as expanded surveys of LYSSAV in Africa (this study and [37]) and in Eurasia [8], [9], [21] reveal greater genetic diversity. More fundamentally, if, as we suggest, complete genomes represent the best tools for genotyping, we propose that 0406SEN should constitute a new GT8 different from GT2 (8619NGA) and that the genotype division should be set at 76.4 to 81.6% nucleotide identity at coding sequences for all five viral proteins. Such a cut-off would provide more discriminatory power than systems that utilize the N gene in isolation (Table 3).

**Table 3:** Minimum intra-genotype and maximum inter-genotype sequence similarities among 24 lyssaviruses.

Coding regions	Number of genotypes	Minimum intragenotype similarity	Maximum intergenotype similarity
**N**	8*	83,3	80,3
	7	80,3	79,8
**N,P,M,G,L**	8*	81,6	76,4
	7	76,3	76,4

* when 0406SEN is considered as the representative isolate of a new GT8, with 8619 the representative isolate of GT2.

Finally, we suggest that the phylogenetic methods used here – based on a realistic model of nucleotide substitution, a robust phylogenetic method, and rigorous bootstrap resampling – represent a more powerful method of lyssavirus classification than those based on pairwise genetic diversity alone, particularly as they account for any lineage-specific rate variation that will compromise all distance-based approaches used to date. This method has also been proposed for HIV to try to standardize viral classification [38] confirming the interest of this method for viral classification.

## Supporting Information

Table S1 List of primers
(0.06 MB PDF)

Table S2 Transcription and termination signals for all lyssavirus genotypes.
(0.05 MB PDF)

Figure S1 Comparison of the 5′ and the reverse complementary 3′ genomic termini of the antigenomic (+) sense RNA of lyssaviruses. Identical nucleotides are indicated by a vertical line. A, 23 lyssaviruses representing the 7 genotypes. B, consensus sequences. Only regions corresponding to 3′ and 5′ UTR sequences are shown. TTS: transcription termination signal.
(0.27 MB DOC)
