# Supplementary material for: Genomic Diversity and Evolution of the Lyssaviruses
Source: PLoS One. 2008 Apr 30;3(4):e2057. doi: 10.1371/journal.pone.0002057 (PMC2327259; doi:10.1371/journal.pone.0002057)
Supplement: Table S1 — List of primers (0.06 MB PDF) [file pone.0002057.s001.pdf]

Table S1: List of primers

Gene	Full name	Primer sequence (5'-->3')[a]
Nucleoprotein		
	N127	F:ATGTAACACCTCTACAATGG
	N540lag	F:AGTTGTAGAGCATCACACTTT
	N9001-600AS	R:TATGGTACTCCAGTTAGCGC
	N9001-420AS	R:AGAGATGCGTGCTCTGAAAC
	N9704-410AS	R:TTCCTTCCACATTAGTACGTC
	N9704-650AS	R:TATAGTACTCCAGTTGGCGC
	N0406-700AS	R:ACAGTCTTCATAAGCAGTCAC
	N0406-510AS	R:CAGTTTCGAAGATCTGCTCC
	N86100-660AS	R:GGTATAGTGCTCCAATTGGC
	N86100-500AS	R:CTATACAGACACAGTAACAGC
	N86101-530AS	R:TTCTGACCAACTATCTTGCTC
	N86101-420AS	R:CCTTCTACATTTGTGCGAGC
	N-03002-667-AS	R:CAGAATACAGGTGCTCAATTC
	N-03002-508-AS	R:TCCATTCTATCTGCCACATTG
	N-8918-580-AS	R:TTAGGTATAGTGCTCCAGTTG
	N-8918-460-AS	R:CCAGTATTCTGTCCTACTATC
	N-9018-570-AS	R:GGCGCACATCTTGTGAGTAG
	N-9018-400-AS	R:AAGATGCATGCTCGGCTGTG
	N-9147-600-AS	R:GTATAGTACTTCCAATTAGCACAC
	N-9147-460-AS	R:TTGCTCAACCTATACAGACTC
	N-94286-570-AS	R:TTGTGAGTGGTCATTAATGTG
	N-94286-500-AS	R:ATCTGTTCCATCCGGTCAGC
	N-Tha-200_AS	R:TCAGGATCAAGCTTGGCAGC
	N-Tha-390_AS	R:TCTCGTCACTTCCATACCTC
	N8619-550AS	R:CAAAGTGTGATGCTCTACAAC
	N8619-410AS	R:AAGTCCTACTAGAGAAGCATG
	N8619-900AS	R:CTGCGTTGGATGAATAAGGAG
	N8619-760AS	R:ATATAAGAGCGCGTCTCGTG
	NLag250-AS	R:TATCCAATCCTCTGGACAC
	NLag500-AS	R:ATCTGCTCCATTCTGTCTGCA
Matrix		
	M220	F:TGGTGTATTCAACATGRAYTC
Glycoprotein		
	G780	R:ACCCATGTYCCRTCCATAAG
	G	F:GACTTGGGTCTCCCGAACTGGGG
	Gh5lag	R:CATTAAAACTGTCTGCTAAAG
Polymerase		
	L1	R:GAGTTNAGRTTGTARTCAGAG
	PVO3	R:CCADMCBTTTTGYCKYARRCCYTC
	PVO5	F:ATGACGGACAAYCTGAACAA
	L11625AS1	R:ATCCAGTGAGATGACARRCT
	L11625AS2	R:ATCCAGTGAGATGACAGACT
	LLag11330-S	F:TTTCCAACAGAGTCTTCAATG
	LLag11560-S	F:TTGGGAAGATCCTGGACCTG
	L-03002-11420-S	F:TATCTATCAGCAGCCTTAGGA
	L-03002-11544-S	F:TGGACAGACAACACTCCAGT
	L-8918-11100-S	F:AAGTGAAATGCTAAGAGCCAG
	L-8918-11200-S	F:CTCATAGACAATGATGTAGAG
	L-9018-11240-S	F:TGTCCATCATCATCGCCATC
	L-9018-11340-S	F:TAAGACACTTCAACATCTGCTG
	L-9147-11440-S	F:TTCACGACCTGTATAACAGAC
	L-9147-11390-S	F:TATGATGTATCTATCTACCGC
	L-94286-11420-S	F:CTCTCTTGTACTTATCAGTTGC
	L-94286-11330-S	F:AGTGTTCAATGTGTCTAAGCC
	L0406-11250S	F:GTTCATAAGATGGTGGATGAG
	L0406-11420S	F:ACATCTGTTCTAGCACACTTC
	L86101-11510S	F:ACAAGACTTCATGAGCTCTAC
	L86101-11430S	F:ATCAGATCCCAAGTTGTTGAG
	L86100-11400S	F:GTCTAAATCTCTGAATGAGCC
	L86100-11460S	F:ACTTCAACATATGTTCCAGTAC
	L9704-11130S	F:CAATTACCAAGATCTTGTAAGG
	L9704-11230S	F:TCTTGGTTCACAAGATGGTAG
	L9001-11430S	F:TTTTATCCTCCATCTGATCCC
	L9001-11300S	F:CACAAGATGGTAGATGATCTAG
	L-Tha-11500S	F:TCCTTCCTGGTTCACAAGATG
	L-Tha-11400S	F:CCTGAGGAGATTATATCTAACC

[a] F, forward; R, reverse
