# Supplementary material for: Genomic Diversity and Evolution of the Lyssaviruses
Source: PLoS One. 2008 Apr 30;3(4):e2057. doi: 10.1371/journal.pone.0002057 (PMC2327259; doi:10.1371/journal.pone.0002057)
Supplement: Table S2 — Transcription and termination signals for all lyssavirus genotypes. (0.05 MB PDF) [file pone.0002057.s002.pdf]

Table S2 Transcription and termination signals for all lyssavirus genotypes.

Gene	Genotype	initiation (a)	termination (b)
**N**	GT1	AACACCYCTACA	TGAAAAAAA CT
	GT2	AACACYMCTACA	AGAAAAAAA CTC
	GT3	AACACTCCTACA	AGAAAAAAA CTC
	GT4	AACACCCCTACA	TGAAAAAAA CT
	GT5	AACACCCCTACA	AGAAAAAAA CT
	GT6	AACACCCCTACA	AGAAAAAAA CT
	GT7	AACACCCCTACA	AGAAAAAAA CT
	Con	AACACYHCTACA	WGAAAAAAA CT-/C
**P**	GT1	AACACCCCTCCT 17 nuc	TGRAAAAAA CARGC
	GT2	AACAYCWCTCCT 17 nuc	TGRAAAAAA CATGC
	GT3	AACACCACTCCT 17/18nuc	TGRAAAAAA CRTGT
	GT4	AACACCACTCCG 17 nuc	TGRAAAAAA CATGC
	GT5	AACACCACTCCG 17 nuc	TGAAAAAAA CATGT
	GT6	AACACCCCTCTT 17 nuc	TGAAAAAAA CATGT
	GT7	AACACCCCTCCT 17 nuc	TGARRAARA CTGTC
	Con	AACAYCHCTCYK 17/18nuc	TGRRRAARA CDDKY
**M**	GT1	AACACCACTGAT 3 nuc	TGAAAAAAA CTWTY
	GT2	AACAYCCCTARA 3 nuc	TGAAAAAAA CTTKTKAAYAMGRWWT
	GT3	AACATCCCTAAA 3 nuc	TGAAAAAAA CTRCATRAAAAGRCWC
	GT4	AACACCACTGAC 3 nuc	TGAAAAAAA CAGGC
	GT5	AACACCACTGAC 3 nuc	TGAAAAAAA CAAAT
	GT6	AACACCACTAAC 3 nuc	TGAAAAAAA CATTT
	GT7	AACACCACTGAT 3 nuc	TGAAAAAAA CAGTT-/T
	Con	AACAYCMCTRRH 3 nuc	TGAAAAAAA CWNNH 0-11 nuc
**G**	GT1	AACAYCCCTCAA 15/16 nuc	RGAAAAAAA C 23 nuc*
	GT2	AACARCCCTCCC 11 nuc	TGAAAAAAA C 25/29 nuc
	GT3	AACAGCCCTCTM 9 nuc	TGAAAAAAA C 27 nuc
	GT4	AACACCCCTCAA 14 nuc	TGAAAAAAA C 35/36 nuc
	GT5	AACATCCCTCGA 14 nuc	TGAAAAAAA C 20 nuc
	GT6	AACATCCCTCAA 14 nuc	TGAAAAAAA C 18 nuc
	GT7	AACAYCCCTCGA 14 nuc	TGARAAAAA C 18 nuc
	Con	AACANCCCTCNM 9/16 nuc	RGARAAAAA C 18-36 nuc
**L**	GT1	AACACTTCTCMT 15/18 nuc	TGAAAAAAA C
	GT2	AACAKYCCTCAT 15 nuc	WGAAAAAAA C
	GT3	AACAMTCCTCCT 15/16 nuc	TGAAAAAAA C
	GT4	AACACCCCTCCT 20 nuc	TGAAAAAAA C
	GT5	AACACCCCTCCT 20 nuc	TGAAAAAAA C
	GT6	AACACCTCTCTT 18 nuc	TGAAAAAAA C
	GT7	AACAYYCCTCCT 18/21 nuc	TGAAAAAAA C
	Con	AACANYYCTCHT 9/21 nuc	WGAAAAAAA C

Con: Consensus.

a. The number of nucleotide (nuc) between the initiation signal and the start codon of the gene are indicated.
b. The number of nucleotide between the termination signal and the initiation of the following gene are indicated.
*PV possesses another termination signal in the G gene – TGAAAAAAA – 422 nucleotides before initiation of the L gene.
