# Supplementary material for: Genomic Diversity and Evolution of the Lyssaviruses
Source: PLoS One. 2008 Apr 30;3(4):e2057. doi: 10.1371/journal.pone.0002057 (PMC2327259; doi:10.1371/journal.pone.0002057)
Supplement: Figure S1 — Comparison of the 5′ and the reverse complementary 3′ genomic termini of the antigenomic (+) sense RNA of lyssaviruses. Identical nucleotides are indicated by a vertical line. A, 23 lyssaviruses representing the 7 genotypes. B, consensus sequences. Only regions corresponding to 3′ and 5′ UTR sequences are shown. TTS: transcription termination signal. (0.27 MB DOC) [file pone.0002057.s003.pdf]

Figure S1 : Comparison of the 5’ and the reverse complementary 3’ genomic termini of the antigenomic (+) sense RNA of lyssaviruses. Identical nucleotides are indicated by a vertical line. A, 23 lyssaviruses representing the 7 genotypes. B, consensus sequences. Only regions corresponding to 3’ and 5’ UTR sequences are shown. TTS: transcription termination signal.

A

```
Genotype 1 :
         5'ACGCTTAACAACAAAATCAGAGAAGAAGCAGACAGTGTCATTTGCAAAGCAAAAATGTAACACCCCTACA N ORF
8743THA    ||||||||||| |||| | | |    | | | |   |||       ||      ||         | |
         3'ACGCTTAACAAAAAAAACAATAAAGATGGAAAAAATAATCAAACAGCTAGAGGTTCTGACTTGAGATTTCGTTTTTTTCACGGTACACTGTCTGAGAATCTTAGTATATACAGATCCACAGAAATCTCCAGC L TTS

         5'ACGCTTAACAACAAAATCAGAGAAGAAGCAGACAGTGTCATTTGCAAAGCAAAAATGTAACACCCCTACA N ORF
8764THA    ||||||||||| |||| | | |  | | | |     |||       ||      ||        || |
         3'ACGCTTAACAAAAAAAACAATAAAGAATGAAAAAGTAATCAAACAGCTAGAGGTTCTGACTTGAGACTTCGTTTTTTTCACGGTACACTGTCTGAGAATCTTAGTCGATACAGATCCTCAGAATTCTCCAGC L TTS

         5'ACGCTTAACAACAAAATCAGAGAAGAAGCAGACAGCGTCAGTTACAAAGCAAAAATGTAACACCCCTACA N ORF
9147FRA    ||||||||||| |||| | | |    | | | |   |||   |   ||       |   ||
         3'ACGCTTAACAAAAAAAACAATAAAGATGGAAAAAACAATCAAACAGCCAGAGGTTCGGATTCAAGATCTTGTTTTTTTCAAGATACATCGCACAAGAGTCTTAGCATGTATAGCCTTCCGGGAGTGCTCGGT L TTS

         5'ACGCTTAACAACAAAACCAGAGAAGAAGCAGACAGCGTCAATTGCAAAGCAAAAATGTAACACCCCTACA N ORF
NNV-RAB-H  |||||||||||  |||| | | |   |  | | |   ||||      ||       |   ||
         3'ACGCTTAACAAATAAACAACAAAGACAAAAAAAACAATCAAACAACCAGAGGCTCAGAGTCAAGATCTTGTTTTTTTCAAGGTATATCGTACAAGAGTATTAGCTTGTACAAGCCTCTGAGGGTGTCCAGT L TTS

         5'ACGCTTAACAACAAAATCAGAGAAGAAGTAGACAGTGTCGTTTACAGAGCAAAAATGTAACACCCCTACA N ORF
SHBRV-18   |||||||||||  |||  | | |   |  | | |   ||    |   ||           ||
         3'ACGCTTAACAAATAAACAACAAAGACAAAAAAAACAATCAAACAACCAGAGGCTCAGAGTCAAGATCTTGTTTTTTTCAAGGTATATCGTACAAGAGTATTAGCTTGTACAAGCCTCTGAGGGTGTCCAGT L TTS

         5'ACGCTTAACAACAAAATCAGAGAAGAAGTAGACAGTATCGTCTACAAAGCAAAAATGTAACACCCCTACA N ORF
9001FRA    ||||||||||| |||| | | |    | | |         |    |             |    |||
         3'ACGCTTAACAAAAAAAACAATAAAGATGGAAAAACAATCGAGCCGACAGAGGTTCGGAATTCTGATCTAGTTTTTTTCAGGGTTCACCGTCCAGGAGTCTCAGTCTGTACATTCTATCAGGGGCACTTGGT L TTS

         5'ACGCTTAACAACAAAATCAGAGAAGAAGTAGACAGTGTCGTCTACAAAGCAAAAATGTAACACCTCTACA N ORF
9704ARG    ||||||||||| |||| | | |||  | | |        ||    |                  ||
         3'ACGCTTAACAAAAAAAACAATAAAGATGGAAAAACAGTCAATCAGACAGAGGTTCTGAATTTGGACCTTGTTTTTTTCAAGGTGTACTGTCCAGGAGTTTTGGTCTGTATATTCCCTTGAGAGTGCACAGT L TTS

         5'ACGCTTAACAACAAAACCAAAGAAGAAGCAGACAGCGTCAATTGCAAAGCAAAAATGTAACACCCCTACA N ORF
RABV       |||||||||||  |||| | | |   |  | | |   ||||      ||       |   ||
         3'ACGCTTAACAAATAAACAATAAAGACAAAAAAAACAATCAAACAACCAGAGGCTCAGAGTCAAGATCTTGTTTTTTTCAAGGTATATCGTATAAGAGTCTCGGTCTGTACAGGCCTCTGAGGGTGTCCAGT L TTS

         5'ACGCTTAACAACCAGATCAAAGAAAAAACAGACAGCGTCAATGGCAGAGCAAAAATGTAACACCTCTACA N ORF
PV         |||||||||||  | |  | | |||  | | | |   ||||      ||       |
         3'ACGCTTAACAAATAAACAACAAAAATGAGAAAAACAATCAAACAACTAGAGGTTCAGATTTAAGATCTTGTTTTTTTCAAAATACA L TTS

         5'ACGCTTAACAACCAGATCAAAGAAAAAACAGACATTGTCAATTGCAAAGCAAAAATGTAACACCCCTACA N ORF
SADB-19    |||||||||||  | |  | | |||  | | | |   ||||      |        |    |
         3'ACGCTTAACAAATAAACAACAAAAATGAGAAAAACAATCAAACAACCAAAGGTTCAGATTTAGGATCTTGTTTTTTTCAAGATACATCACACAAGAGTCTTAGCATGGGCAGGCTTCCAGGAGTATCCGGT L TTS
Genotype 2 :
         5'ACGCTTAACAACAAAATCAGAGAAGACATAGACACCTACAATGAGGTAATCAAAATGTAACACTACTACA N ORF
8619NGA    ||||||||||| |||| |  | || |  || | ||   ||    | |   |     |  ||      |
         3'ACGCTTAACAAAAAAAAACGAATAAAAATTAAAAACAATCACATGGAGAGACGTTCAGACACTTGCAGATGTTTTTTTCTATATTTTCTCTTTGGTCTGTGTATCACATTTAGAGAGAGGAAAAAGGTCAAAATTCGACAACATTT L TTS

         5'ACGCTTAACAGCAAAATCAGAGAAGATATAGACAGCGACAATGCGCTGAACAAAATGTAACACCCCTACA N ORF
0406SEN    ||||||||||  ||||  | | ||||  || | |  | |||        ||     | |||   |  |
         3'ACGCTTAACAAAAAAAACAATACAAGAATTAAAAACAGTCAAGTTAAGATACTCTCAGAAACTTGCAGATGTTTTTTTCACTGTAAATTCTTTGGCATGTATCAGTCACTTTCACCTAGTACACAGTTTGTCATCCCGAAGCTCTT L TTS
Genotype 3 :
         5'ACGCTTAACAACAAAATCAAAGAAGACATAGACAGTATCAGCGACCTAAACAAAATGTAACACTCCTACA N ORF
86100CAM   ||||||||||| |||| || | || |  || | |  ||||       |  || |||||||   |
         3'ACGCTTAACAAAAAAAACCATATAAAAATTAAAAACAATCACATGATGATGCATAATGTAATTGTTACTTGTTTTTTTCAATGATGCTCTCACAGCTTATCCATATTGATTGGA L TTS

         5'ACGCTTAACAACAAAATCAAAGAAGACATAGACAGTAGCAGTGACCTAAACAAGATGTAACACTCCTACA N ORF
86101RCA   ||||||||||| |||| || | || |  || | |  | ||  |  | |  || |||||     |    |
         3'ACGCTTAACAAAAAAAACCACATAAAAATTAAAAACAATCACAGGACAATGCATGATGTGCTTGTTACTCGTTTTTTTCACGAATGCTTTTTAATCTATCAAAATAGAACTGA L TTS

        5'-ACGCTTAACAACCAGATCAAAGAAGACACAGATAGTATCAGTGACCTAAACAAAATGTAACACTCCTACA N ORF
MOKV       |||||||||||  | | || | || |  || | |  ||||  |  | |  || || ||||
        3'GACGCTTAACAAAAAAAACCATATAAAAATCAAAAATAATCACAGGACAATGCATAAGGTAATTGCTACCTGTTTTTTTCAATGATGCTCTCCAAGTCTATCACCATTGATTAGA L TTS
Genotype 4 :
         5'ACGCTTAACAACAAAATCATAAAAGGGGCAGACATGTTCATTTGTATAACACAAATGTAACACCCCTACA N ORF
86132SA    ||||||||||| |||| || | |||   || |     |||   |   |  |  |  | |     |  |
         3'ACGCTTAACAAAAAAAAACAAACAAGACACAAAATGAGTCAAGGGGGCAGAAGTACAGAATGTTTCAAATGTTTTTTTCATGTTCTCTGGTATGGCTTTTGGGGACATATATAATATTATGGTCAGAGATAC L TTS

         5'ACGCTTAACAACAAAATCATAAAAGGGGCAAACATGTTCATTTGTATAACACAAATGTAACACCCCTACA N ORF
94286SA    ||||||||||| |||| || | |||   ||||     |||   |   |  |  |  | |     |  |
         3'ACGCTTAACAAAAAAAAACAAACAAGACACAAAATGAGTCAAGAGGGCAGAAGTACAGAATGTTTCGAATGTTTTTTTCATGTTCTCTGGTATGGCTTTTGGGGACATATATGATATTATGGTCAGAGATAC L TTS
Genotype 5 :
         5'ACGCTTAACAACAAAAACAAAAAAGGGGTAGACACGTTCATCGATAGAACAGAAATGTAACACCCCTACA N ORF
RV9        ||||||||||| ||||||||||||     |||      ||   ||  || |||   | |
         3'ACGCTTAACAAAAAAAAACAAAAAAAACAGAGAATGAGCCAAAAATGTAAAAGACCAGGATGAAGGAGATGTTTTTTTCACATCTCTTGACTCGACTAGGCCACAGTGTGAAAAGCATGTTGACAGATGGGA L TTS

         5'ACGCTTAACAACAAAAACAAAAAAGGGGTAGACACGTTCATCGATAGAACAGAAATGTAACACCCCTACA N ORF
03002FRA   ||||||||||| ||||||||||||     |||      ||   ||  || |||   | |  |     |
         3'ACGCTTAACAAAAAAAAACAAAAAAAACAGAGAATGAGCCAAAAATGTAAAAGACCAGGATGAAAGAAATGTTTTTTTCACATCTCTTGGTTCAACTAGGCCACAGTGTGAAAAGCATGTTGACAGATGGGA L TTS

         5'ACGCTTAACAACAAAATCAAAAAAGGGGTAGACACGTTCATCGATAGAACAGAAATGTAACACCCCTACA N ORF
8918FRA    ||||||||||| |||| |||||||     |||   |   |       ||       |    |
         3'ACGCTTAACAAAAAAAAACAAAAAAAACAGAGATGAGCCAAAGATGTAAAAGACCCGGATGAAGGAGATGTTTTTTTCACATTTCTTGATTCGACTAGGCTACAGTGTGGAAGGCATGTTGACGGATAGGA L TTS
Genotype 6 :
         5'ACGCTTAACGACAAAACCAGAAAAGGAATAGACACAATCGTCTGTAGAGCAGAAATGCAACACCCCTACA N ORF
9018HOL    ||||||||| | |||| || | ||  || |    ||  |        |       ||
         3'ACGCTTAACAAAAAAAAACATAGAAAAAAGAAGACCATCCAAACAGGTACATATCCTGACTTCAGATATTGTTTTTTTCACGCCCATTTTCCTTAGGGAAATTGCTGTTTCATAAGTGGCACATGTCCTGCC L TTS

         5'ACGCTTAACGACAAAACCAGAAAAGGAATAGACACAATCGTCTGTAGAGCAGAAATGCAACACCCCTACA N ORF
RV1333     ||||||||| |||||| || | ||  || |    ||  |        |       ||
         3'ACGCTTAACAAAAAAAAACATAGAAAAAAGAAGACCATCCAAACAGGTAAATATCCTGACTTCAGATATTGTTTTTTTCACGCCCATTCTCCTTAGGGAAATTGCTGTTTCATAAGTGGCACATGTCCTGCC L TTS
Genotype 7 :
         5'ACGCTTAACGACAAAACCAGAGAAGGAGTAGACATGATCATTTGCGAAGCAAAAATGTAACACCCCTACA N ORF
ABLh       ||||||||| | |||| |  | ||| | || | |   |||     | |       || | |   | ||
         3'ACGCTTAACAAAAAAAAACCAACAAGAAATAAAAACATTCAAACAAGTACAGGTTCTGAATCTTGCATATGTTTTTTTCAAGCCTCTCGCACCAAACATGATTTCAATAGAGAAGACGCTGGAGGATCCCAA L TTS
```

B

```
All genotypes :
        5'ACGCTTAAC--C-A-A
Consensus |||||||||    | |
        3'ACGCTTAACAAA-AAA
```
